# Short-Term Exposure to Violet Light Emitted from Eyeglass Frames in Myopic Children: A Randomized Pilot Clinical Trial

**DOI:** 10.3390/jcm11206000

**Published:** 2022-10-11

**Authors:** Hidemasa Torii, Kiwako Mori, Takashi Okano, Shinichiro Kondo, Hao-Yung Yang, Erisa Yotsukura, Akiko Hanyuda, Mamoru Ogawa, Kazuno Negishi, Toshihide Kurihara, Kazuo Tsubota

**Affiliations:** 1 Department of Ophthalmology, Keio University School of Medicine, 35 Shinanomachi, Shinjuku-ku, Tokyo 160-8582, Japan; 2 Laboratory of Photobiology, Keio University School of Medicine, 35 Shinanomachi, Shinjuku-ku, Tokyo 160-8582, Japan; 3 Smile Eye Clinic, Kanzeme Building 4F, 1-6-12 Aoba-ku, Yokohama 227-0062, Japan; 4 Tsubota Laboratory, Inc., 304 Toshin Shinanomachi-ekimae Building, 34 Shinanomachi, Shinjuku-ku, Tokyo 160-0016, Japan

**Keywords:** myopia, violet light, randomized double-blind placebo-controlled clinical trial, safety, efficacy

## Abstract

Violet light (VL), 360–400 nm wavelength, is contained in the sunlight and is an effective element for myopia suppression. This study is to investigate the safety and efficacy of novel eyeglasses that emit VL from the frames. This is a double-masked, randomized, pilot clinical trial conducted in a clinic in Japan. Forty-three children with myopia were enrolled. Participants were randomly assigned to two groups, wearing VL-emitting eyeglass frames (VLf) that emitted VL of 310 μW/cm^2^ (VLf group, *n* = 22) or pseudo-placebo eyeglass frames with a minimal emission of VL (<10 μW/cm^2^) (control group, *n* = 21). The exposure time was 3 h per day. The primary outcomes were visual acuity, tear film break-up time, corneal endothelial cell density, and the slit-lamp/fundus examinations. The secondary outcome was the 6-month changes in the axial lengths and cycloplegic refractions. Forty-one (95%) participants were included; twenty-one in the VLf group and twenty in the control group. No significant differences were seen in any safety evaluation. Significant changes were seen in axial elongation, choroidal thickness, and cycloplegic refractions in the subgroup analysis of 8- to 10-year-old children (*p* < 0.05), but otherwise no significant differences were seen. The VLf showed short-term safety and effectiveness against myopia progression.

## 1. Introduction

The upsurge in myopia worldwide [1], and especially in East Asia [2,3], is well known. This trend started only during the past few decades [2], which implies the importance of environmental factors involved in the onset and progression of myopia. Epidemiologic studies have suggested that increased near visual tasks such as reading and using computers and smartphones are possible risk factors [4]. However, the time spent outdoors is an important protective factor against myopia [4,5,6,7,8,9,10,11,12]. In addition, some clinical trials have indicated that increased outdoor activity by students had a preventive effect against myopia [12,13,14].

Wu et al. [14] reported that the Taiwanese government decreased the prevalence of myopia in Taiwan by introducing mandatory outdoor activities for at least 120 min daily. They also showed that relatively lower outdoor light intensity, such as that in the shade of a tree, was sufficiently strong to suppress myopia progression [15], while the beneficial effect of high ambient light to protect against myopia has been confirmed in chicks, mice, and monkeys [16,17,18,19,20]. These results imply the importance of the wavelength of sunlight including violet light (VL) against the progression of myopia in humans.

VL, with wavelengths from 360 to 400 nm, is abundant outdoors [21]. We reported that VL was effective in chicks [21], mice [22], and humans (children [21,23] and adults [24]) against myopia progression. The protective effect of VL on myopia progression required retinal expression of the VL-sensitive atypical opsin, neuropsin (OPN5), and Opn5-expressing retinal ganglion cells with VL prevented experimental myopia in mice [22].

Although the outdoor environment is crucial to protect against myopia [25], individuals tended to remain indoors due to the coronavirus pandemic. Wang et al. [26] reported a significant myopic shift for children aged 6 to 8 years due to home confinement. The Tsubota Laboratory, Inc. (Tokyo, Japan) in collaboration with NEWOPTO CORPORATION (Kanagawa, Japan) devised a novel device that emits VL from eyeglass frames (VLf) to suppress myopia progression with both indoor and outdoor use (patent no. 6175210).

The main objective of the current study was to confirm the safety of VLf and the secondary objective was to observe whether the eyeglasses slowed myopia progression in a double-masked, randomized, pilot clinical trial with 6 months of use.

## 2. Materials and Methods

### 2.1. Study Oversight

The Medical Corporation Shintokai Yokohama Minoru Clinic Institutional Review Board and the Keio University School of Medicine Ethics Committee approved the study protocol (approval numbers, 6019-145-22S487 and 20210148, respectively). All procedures involving human subjects were performed in accordance with the tenets of the Declaration of Helsinki. Written informed consent was obtained from all patients and their guardians after they received an explanation of the study. A monitoring committee oversaw the trial and reviewed the trial data. This clinical study was registered at the UMIN Clinical Trial Registry (first trial registration on 09/04/2019, UMIN 000036453).

### 2.2. Study Objective, Design, and Setting

The first and secondary objectives of this randomized, double-blind, pseudo-placebo-controlled, parallel group comparative study, respectively, were to confirm the safety of the VL emitted from the eyeglass frames and to observe whether the eyeglasses slowed myopia progression by evaluating the cycloplegic objective and subjective refractions and axial length prospectively. The choroidal thickness was separately and retrospectively evaluated.

The trial was conducted at the Smile Eye Clinic, Yokohama, Japan. The participants were enrolled between April and June 2019 (initial subjects) and between September 2019 and January 2020 (subsequent subjects). The trial was suspended after the first period because of the fragility of the eyeglass frame. The trial resumed after the frame was upgraded with a stronger material. The follow-up period was completed on 7 July 2020.

The inclusion criteria were as follows: patient age of 6 to 12 years; a cycloplegic objective refraction of each eye between −1.50 diopters (D) and −4.50 D; anisometropia of 1.50 D or less; astigmatic values of ±1.50 D or less in each eye; a corrected visual acuity (VA) of 20/20 or better in each eye; and at least one myopic parent.

The exclusion criteria were: a history of ocular surgery, epilepsy, amblyopia, or manifest strabismus; use of psoralens; confirmed or suspected photosensitivity; binocular dysfunction; 1.50 D or greater binocular difference in objective refraction between both the cycloplegic and noncycloplegic refractions; cornea guttata or corneal endothelial cell density of less than 2000 cells/mm^2^; current or past use of drugs affecting the neurotransmitters or growth; treatment with atropine, orthokeratology, multifocal eyeglasses, or progressive power lenses; a history of allergies to fluorescein, oxybuprocaine, cyclopentolate hydrochloride, tropicamide/phenylephrine hydrochloride, or benzalkonium; and another family member already participating in the trial.

For the safety evaluation, the sample size was 40 cases (2 groups of 20 cases/group).

### 2.3. Randomization and Double Masking

The subjects were assigned randomly and equally to the trial and control devices so that the ratio of the trial to control devices was 1:1. The randomization assignment was stratified by the size of the eyeglasses (large or small) using a random permuted block design with varying block sizes of 4. The data coordinating center verified the appropriate treatment group assignment after the unmasked examiner enrolled each participant. Masked examiners performed all measurements at every visit.

**Figure 1:**
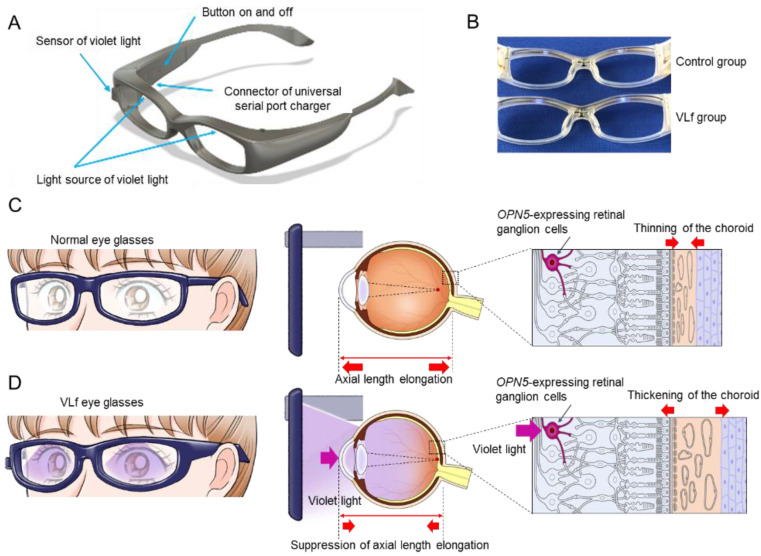
The schema and mechanism of the VLf that emits violet light (VL). The pseudo-placebo (control) and VLf (**A**). The control eyeglasses emit a minimal irradiance of VL under 10 µW/cm^2^ (control group, top of **B**) and VLf emit VL irradiance of 310 µW/cm^2^ (VLf group, bottom of **B**). In the myopic patients with normal eyeglasses, the axial length is elongated with choroidal thinning (**C**). In the myopic patients with VLf, the axial length elongation is suppressed with choroidal thickening, presumably through OPN5-expressing retinal ganglion cells by emitting VL from the VLf frames, as was demonstrated in the mouse experience (**D**). VLf: violet light eyeglasses frames.

Regarding the control group, the participants could have readily noticed if the eyeglasses emitted no light. Therefore, although the frames were identical in both groups (Figure 1A), we made pseudo-placebo eyeglasses for the control group with a minimal amount of VL irradiance (<10 µW/cm^2^) to maintain the double-blind nature of the study (Figure 1B). The participants and parents/legal guardians were masked by removing all eyeglass labels before they received the eyeglasses.

### 2.4. Intervention

The VL was irradiated for 3 h daily in both groups from 11 am to 2 pm in consideration of the effect on circadian rhythm. Because over 2 h of daily outdoor activity suppresses myopia progression [8,9,14], we set the daily exposure to 3 h.

The annual average VL irradiance in Tokyo is 310 μW/cm^2^, based on our measurement data and the climate statistics available in “Statistical Observations of Prefectures” by the Statistical Bureau of Japan (http://www.stat.go.jp/data/ssds/index.html (accessed on 1 August 2017)). We set the device to this irradiance value, which meets domestic Japanese Industrial Standards and International Electrotechnical Commission safety standards. The specifications are summarized in Table S1 (Supplementary Materials).

A VL sensor was attached to the VLf eyeglass frames (Figure 1A) for both the test and pseudo-placebo groups to assess the accurate VL exposure while outdoors.

The adherence to eyeglass use was determined through VL irradiation time records on a cloud server, saved via the VLf, and software installed on an iPod touch (Apple Inc., Cupertino, CA, USA).

### 2.5. Mechanism of Action of the Study Eyeglasses

Myopic patients wearing ordinary eyeglasses have an elongated axial length and thinned choroid (Figure 1C). However, with the VL emitted from the study eyeglasses, the axial elongation in the myopic eyes was suppressed with a thickened choroid through OPN5-expressing retinal ganglion cells (Figure 1D) [22].

### 2.6. Outcomes

The primary outcome was a safety evaluation that compared the VA, intraocular pressure measured using non-contact tonometer (FT-01; Tomey, Nagoya, Japan), tear film break-up time (BUT), corneal endothelial cell density measured using specular microscope (EM-3000; Tomey, Nagoya, Japan), findings on slit-lamp/fundus examinations, retinal morpho-structural evaluation using optical coherence tomography (OCT) (DRI OCT Triton; Topcon, Tokyo, Japan), dermopathy (periocular skin), and adverse events. Scoring of the ocular surface was performed with 1% fluorescein dye. The fluorescein corneal and conjunctival staining scores of the ocular surface ranged between 0 and 9 points as we reported previously [27].

The secondary outcomes were the 6-month changes in axial lengths measured using partial interferometry biometry (IOLMaster 700; Carl Zeiss Meditec AG, Jena, Germany) and cycloplegic refractions measured using corneal topographer (RT-7000; Tomey, Nagoya, Japan), recorded as the mean of five measurements, from screening up to 6 months at each visit. We used a linear mixed-effects model and compared the changes in myopia progression and axial elongation between the two groups. We also evaluated the choroidal thickness retrospectively using OCT (DRI OCT Triton; Topcon, Tokyo, Japan) as reported previously [28].

After the examinations, the patients and guardians completed a questionnaire that included the recording of age [29,30], sex [31], parental myopia [9], time spent in outdoor activity before entering elementary school [25], time spent performing near work [32], reading distance [4], time spent sleeping [33,34], club activity (outdoors/indoors) [35], and time exposed to sunlight [14,15], all of which are known to be confounding environmental factors regarding myopia progression.

We conducted follow-up examinations at 4, 12, and 24 weeks after the first visit. If the VA with the current spectacles decreased below 20/20, the lens was replaced based on the cycloplegic subjective refraction.

### 2.7. Adverse Events

An adverse event is any undesired medical occurrence experienced by the subjects, regardless of their causal relationship with the device. Any adverse event that met one of the following criteria was considered serious: a fatality; a life-threatening episode; requirement for hospitalization or prolonged hospitalization; a resulting disability; cause of a birth defect; and any serious event associated with the previous factors.

### 2.8. Statistical Analyses

The *t*-test was used to compare the baseline data. The chi-square test was used for categoric variables. The Mann–Whitney U-test was used to compare changes in the choroidal thickness. Statistical significance was defined as *p* < 0.05.

Regarding the primary outcomes (safety; Table S2), a mixed-effects model for repeated measures (MMRM) was used with a variance–covariance structure of repeated data postulating unstructured. The participant identification number was included as a random effect in this model. The fixed effects in this model were the treatment group, eye, visit, baseline data, and interaction of group and visit.

Regarding the secondary outcomes (efficacy; Tables S3–S5), the null hypothesis was that there would be no difference in myopia progression after 24 weeks between the two groups. In a MMRM model, we used all of the measurements obtained from both eyes of each participant. The participant identification number was included as a random effect. A restricted maximal-likelihood estimation method was used to calculate the estimator. The fixed effects in this model were the treatment group, eye, visit, baseline data, outdoor VL exposure, and questionnaire items (age, sex, parental myopia, baseline objective variables (axial length or cycloplegic objective refraction or cycloplegic subjective refraction), intraocular pressure, fluorescein corneal and conjunctival staining score, BUT, time spent in outdoor activity before entering elementary school, time spent performing near work, reading distance, time spent sleeping, club activity, time of sunlight exposure), and interaction of group and visit. The correlation of repeated measurements was specified for each visit regarding the right and left eyes.

The least-squares means difference and standard error of changes from baseline in the axial length and the cycloplegic objective and subjective refractions with MMRM between the groups and visit were calculated and compared using the *t*-test. We calculated the partial regression coefficient, the 95% confidence interval, and *p* values from the model (Tables S3–S8).

All statistical analyses were performed using SAS 9.4 Foundation for Microsoft Windows for x64 (SAS Institute Inc., Cary, NC, USA).

## 3. Results

**Figure 2:**
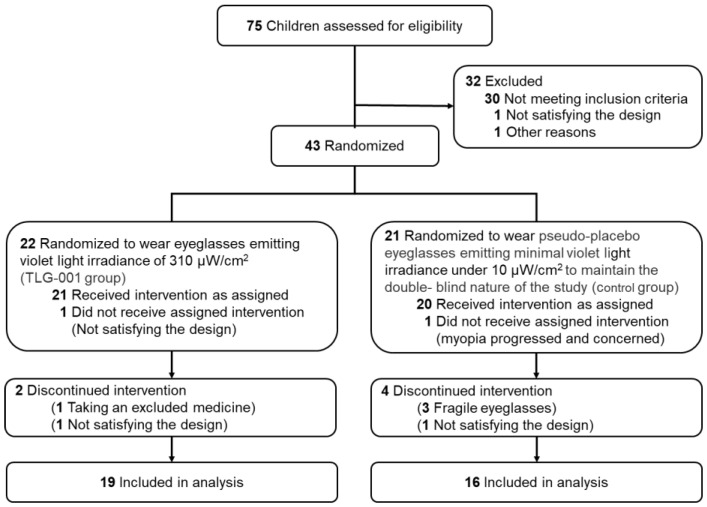
Flowchart of this double-blind randomized pilot clinical trial time points and number of participants.

Of the 75 children assessed, 43 were enrolled and randomized, 2 did not receive the assigned intervention, 41 were included in the analyses (Figure 2), and 35 (85.4%) completed the 6-month visit. Apart from the BUT, no significant differences were found between the two groups in the baseline data and environmental factors (Table 1).

### 3.1. Safety and Effectiveness Evaluations

No tendency was observed for any change in the numerical values for the right, left, and both eyes during the visits for both groups (Table S2). Further, no significant differences were observed between the two groups in the changes in these items from baseline to the 4-, 12-, and 24-week visits (Table S2). No abnormalities were observed in the slit-lamp microscopic and fundus examinations, retinal morpho-structural evaluation using OCT, or dermopathy.

No significant differences were observed in the changes in the axial length, cycloplegic objective refraction, and cycloplegic subjective refraction at 24 weeks.

### 3.2. Subgroup Analysis

Participants were separated into groups based on their inclusion in the initial subjects (22 eyes/11 cases) and the subsequent subjects (60 eyes/30 cases) of the study. Considering possible seasonal variations in myopia progression [36,37,38,39], we analyzed the subgroup data. The subsequent subjects, excluding discontinued interventions (56 eyes/28 cases), were separated into the following subgroups; 6- to 7-year-olds (*n* = 2 and 2 eyes in the VLf and control groups), 8- to 10-year-olds (*n* = 10 and 20 eyes), and 11- to 12-year-olds (*n* = 14 and 8 eyes), as described in the statistical analysis report before the start of the trial.

**Figure 3:**
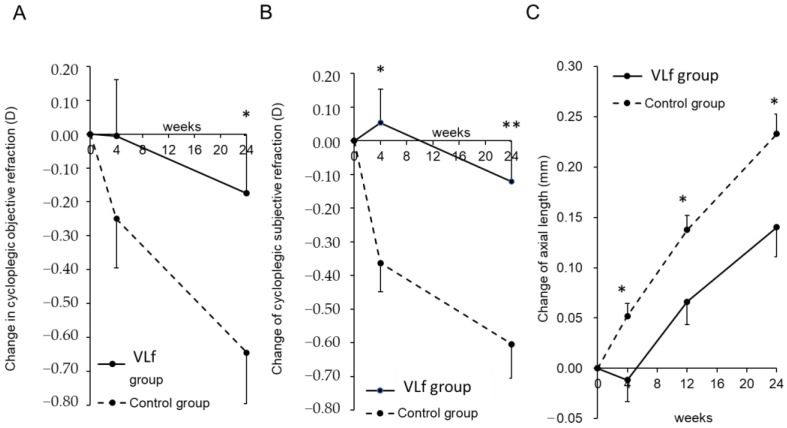
Time course of the changes in the adjusted mean cycloplegic objective and subjective spherical equivalent refractions (SER) and axial length elongation as a result of subgroup analysis in the subsequent subjects aged 8 to 10 years (*n* = 30 eyes comprised of 10 and 20 eyes in the VLf and control groups, respectively). The results were obtained using a linear mixed-effects model analysis. The adjusted mean change in the cycloplegic objective SER in the VLf group is significantly (*p* = 0.048) smaller than that in the control group at 24 weeks (**A**). The adjusted mean changes in the cycloplegic subjective SER in the VLf group are significantly (*p* = 0.012 and *p* = 0.008) smaller than in the control group at 4 and 24 weeks, respectively (**B**). The adjusted mean changes in the axial length elongation in the VLf group are significantly (*p* = 0.020, *p* = 0.015, and *p* = 0.016) smaller than in the control group at 4, 12, and 24 weeks, respectively (**C**). The black lines indicate the VLf group; the dotted lines indicate the control group. The error bars indicate the standard errors. * *p* < 0.05, ** *p* < 0.01. D, diopters. VLf: violet light eyeglasses frames.

**Figure 4:**
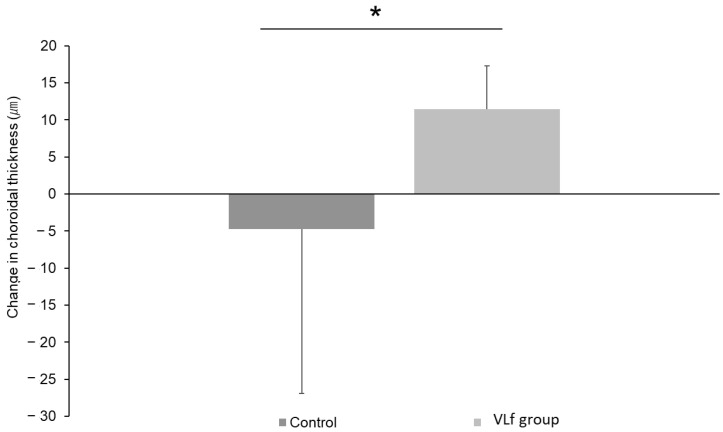
The change in the choroidal thickness at 24 weeks in the subsequent subjects aged 8 to 10 years (*n* = 30 eyes comprised of 10 and 20 eyes in the VLf and control groups, respectively). The change in the choroidal thickness in the VLf group is significantly (*p* = 0.014) larger than in the control group. * *p* < 0.05, Mann–Whitney U-test. VLf: violet light eyeglasses frames.

Subgroup analysis of the MMRM in the subsequent subjects aged 8 to 10 years indicated that the change in the cycloplegic objective refraction in the VLf group was significantly (*p* = 0.048) smaller than in the control group at 24 weeks (Figure 3A, Tables S3–S8 (Supplementary Materials)). The changes in the cycloplegic subjective refraction in the VLf group were significantly (*p* = 0.012 and *p* = 0.008) smaller than in the control group at 4 and 24 weeks, respectively (Figure 3B, Tables S3–S8 (Supplementary Materials)). The axial length elongation in the VLf group was significantly (*p* = 0.020, *p* = 0.015, *p* = 0.016) smaller than in the control group at 4, 12, and 24 weeks, respectively (Figure 3C, Tables S3–S8 (Supplementary Materials)). In the subsequent subjects aged 8 to 10 years, the choroid in the VLf group was significantly (*p* = 0.014) thicker than in the control group (Figure 4).

Subgroup analysis of the MMRM in the initial subjects aged 6 to 7 years, 8 to 10 years, and 11 to 12 years, and in the subsequent subjects aged 6 to 7 years and 11 to 12 years showed that the changes in the axial length and the cycloplegic and subjective refractions did not converge.

### 3.3. Adverse Events

Twelve adverse events were reported throughout the trial. In the VLf group, the following were reported: two cases of nasopharyngitis and one case each of influenza, abdominal pain, nausea, erythema on the skin of the nose in contact with the nose pad of the eyeglass frame and fracture of the upper limbs (including duplicate cases). In the control group, three cases of nasopharyngitis and one case each of influenza, molluscum contagiosum, parotitis, streptococcal infection, sudden deafness, application site erythema, upper limb fracture, and eyelid contusion were reported (including duplicate cases) (Table 2). A comparison of the 95% confidence intervals for each group showed no difference between the groups (Table S2 (Supplementary Materials)).

Two adverse events for which a causal relationship could not be ruled out were erythema on the skin of the nose in contact with the nose pad on the eyeglass frame in one case in each group. The erythema resolved after the fit of the frames was adjusted at the spectacle store. We concluded that erythema was not a phenomenon peculiar to the device or caused by VL.

## 4. Discussion

In this 6-month randomized, double-masked, pilot clinical trial, VLf was not associated with any safety concerns regarding VL irradiation for 3 h daily for 24 weeks. While there were no significant differences between the two groups in the changes in the axial length and the cycloplegic objective and subjective refractions, our subgroup analysis showed significant differences in these parameters.

Many confounding environmental factors are associated with myopia including outdoor VL exposure [21,22,24], time of outdoor activity before entering elementary school [25], time spent performing near work [32], reading distance [4], time spent sleeping [33,34], club activity [35], average time of sunlight exposure [14,15], intraocular pressure [40,41], and dry eye [3,42]. We used these factors as fixed effects for the subgroup analysis of the MMRM in the subsequent subjects aged 8 to 10 years, which was the largest subgroup in the current study; the axial elongation and myopia progression were suppressed significantly in the VLf group compared with the control group. Although these are the exploratory results without adjustment for multiple comparisons, the suppressive rates of axial elongation and myopia progression (cycloplegic objective, subjective refraction) were 40.0%, 72.9%, and 80.0%, respectively. The current results were similar to those of Ho et al. [43], who reported a 60% suppressive effect resulting from 2 h of outdoor activity. The strength of the current study was the outdoor VL exposure measured by the VL sensor, which was then used for the analysis. We believe that the carefully adjusted confounding factors and accurately measured VL exposure helped to achieve the high myopia suppression rate. The progression rate of myopia in the control group of this study demonstrated as 0.65 D in 6 months, which would correspond to 1.30 D in a year, seems faster than other studies for Asian children. However, this value could be reasonable for controls in Japanese children when compared to preceding studies performed in Japan showing the speed of progression was 0.67 D in 6 months reported by Mori et al. [44].

The axial elongation in myopic children using the VLf eyeglass frame was actually suppressed with thickening of the choroid, the mechanism of which was presumably through OPN5-expressing retinal ganglion cells by emitting VL from the VLf frame, as demonstrated in the animal study. Furthermore, the change in the choroidal thickness in the VLf group was significantly larger than in the control group. Choroidal thinning resulting from accommodation is the key mechanism of myopia that causes ocular ischemia [45]. Wu et al. [45] reported that HIF-1α in the sclera was upregulated in the myopia model, which in turn induced the collagen remodeling resulting in scleral weakening, and ocular elongation then occurred. This ischemia, i.e., HIF-1 up-regulation in the sclera, is caused by ischemia of the choroidal blood perfusion [46]. It has been reported that myopia is characterized by a thin choroid, and highly myopic patients have an especially thin choroid [47]. Recent studies [47,48] have shown that choroidal thinning occurs first before eye elongation. The maintenance of the choroidal blood flow and choroidal thickness is fundamentally therapeutic for preventive of myopia. Jiang et al. [22] reported that the non-visual photoreceptor OPN5 stimulation by VL led to maintenance of the choroidal thickness in the murine model of myopia. In this study, the axial elongation was suppressed with choroidal thickening in the VLf wearers, which means that the choroidal blood flow was maintained and ischemia was at least partially resolved, as demonstrated in the preceding studies in animals as well as humans. The VL emitted from the eyeglass frames can be a fundamental therapeutic modality to suppress myopia.

### Limitations

The current study had some limitations. First, the sample size was not large; this was a pilot study to evaluate the safety of VL. Second, the secondary outcome was the exploratory results without adjustment for multiple comparisons. Lastly, the quality of vision during the violet light exposure was not assessed in this study, though it may have been interfered with by wearing VLf. In order to reach a more definite conclusion on the VLf, studies with larger samples and longer follow-up should be conducted.

## 5. Conclusions

The VL emitted from the VLf eyeglass frame was safe in the short-term and partially effective against myopia progression.

## Figures and Tables

**Table 1:** Characteristics of the 82 eyes of 41 participants.

Characteristic	Category	VLf	Control	*p*–Value	
Number of cases		21	20		
Number of eyes		42	40		
Age (years)		9.8 ± 1.7	9.6 ± 1.3	0.588	†
Sex	Male	15 (71.4%)	11 (55.0%)	0.275	††
	Female	6 (28.6%)	9 (45.0%)		
Parents with myopia	Both parents	14 (66.7%)	15 (75.0%)	0.473	††
	Only father	5 (23.8%)	2 (10.0%)		
	Only mother	2 (9.5%)	3 (15.0%)		
Uncorrected visual acuity (log MAR)		0.81 ± 0.23	0.86 ± 0.25	0.312	†
Best corrected visual acuity (log MAR)		−0.09 ± 0.03	−0.08 ± 0.03	0.291	†
Cycloplegic subjective refraction (SE, diopters)		−2.41 ± 1.01	−2.40 ± 0.83	0.97	†
Cycloplegic objective refraction (SE, diopters)		−2.53 ± 0.99	−2.57 ± 0.80	0.853	†
Axial length (mm)		24.58 ± 0.61	24.59 ± 0.75	0.928	†
Intraocular pressure (mmHg)		17.1 ± 2.6	17.5 ± 2.5	0.482	†
Tear film breakup time (seconds)		7.0 ± 2.2	6.0 ± 1.2	0.014 *	†
Fluorescein corneal and conjunctival		0.1 ± 0.3	0.1 ± 0.3	0.951	†
staining score					
Corneal endothelial cell density		2917 ± 207	2940 ± 226	0.634	†
(cells/mm^2^)					
**Environmental factors**					
Time of near work (min/day)		88.8 ± 89.2	59.3 ± 59.1	0.221	†
Time of sunlight exposure (min/day)		52.9 ± 41.2	64.0 ± 68.4	0.527	†
Time of sleeping (min/day)		520.0 ± 41.7	533.5 ± 36.7	0.279	†
Distance of reading (cm)		24.6 ± 7.8	24.8 ± 6.6	0.962	†
Club activity	Outdoor	12 (57.1%)	15 (75.0%)	0.2	††
	Indoor	8 (38.1%)	4 (20.0%)		
	None/unknown	1 (4.8%)	1 (5.0%)		
Time of outdoor activity	0≤ <30	0 (0%)	0 (0%)	0.522	††
before entering elementary school					
(min/day)	30≤ <60	2 (9.5%)	1 (5.0%)		
	60≤ <120	9 (42.9%)	6 (30.0%)		
	120≤	10 (47.6%)	13 (65.0%)		

Data represent means ± standard deviations; log MAR: Logarithm of the Minimum Angle of Resolution; VLf: violet light eyeglasses frames; SE: spherical equivalent; †: *t*-test; ††: Chi-square test; *: statistically significant.

**Table 2:** Evaluation of safety with equipment of VLf or placebo frames.

No. of Subjects	41 Patients	VLf (*n* = 20)	Control (*n* = 21)
Associated adverse reactions	No. of subjects	0	0
	Conjunctival hyperemia	0	0
	Corneal abnormality	0	0
	Lens opacity	0	0
	Inflammation of anterior chamber	0	0
	Fundus abnormality	0	0
	Abnormal findings in OCT	0	0
	Periorbital skin abnormality	0	0
Adverse events	No. of subjects	11	7
	Influenza infection	1	1
	EB virus infection	1	0
	Streptococcal infection	1	0
	Epipharyngitis	3	2
	Mumps	1	0
	Sudden deafness	1	0
	Nausea	0	1
	Abdominal pain	0	1
	Application site erythema	1	1
	Fracture	1	2
	Eyelid contusion	1	0

VLf: violet light eyeglasses frames; OCT: optical coherence tomography.

## Data Availability

The datasets generated and analyzed during the current study are not publicly available. Verification of the clinical trial is currently in progress and the data could not be released to the public until it is finished. However, they are available from the corresponding author on reasonable request.

## Supplementary Materials

The following supporting information can be downloaded at: https://www.mdpi.com/article/10.3390/jcm11206000/s1, Table S1. Specifications of VLf.; Table S2. Safety results regarding visual acuity, intraocular pressure, fluorescein corneal and conjunctival staining scores, tear film break-up time, and corneal endothelial cell density; Tables S3–S5. Results of the mixed-effects model fitted to the 6-month changes in the cycloplegic objective/subjective refractions and axial length in both eyes in the subgroup analysis (second-half cases aged 8 to 10 years; *n* = 30 eyes comprised of 10 and 20 eyes in the VLf and placebo groups, respectively); Tables S6–S8. The adjusted mean changes in the cycloplegic objective and subjective refractions and axial length in both eyes at each visit in the subgroup analysis (second half cases aged 8 to 10 years; *n* = 30 eyes comprised of 10 and 20 eyes in the VLf and placebo groups, respectively).
