# Supplementary material for: Short-Term Exposure to Violet Light Emitted from Eyeglass Frames in Myopic Children: A Randomized Pilot Clinical Trial
Source: J Clin Med. 2022 Oct 11;11(20):6000. doi: 10.3390/jcm11206000 (PMC9604831; doi:10.3390/jcm11206000)
Supplement: Supplementary file 1 [file jcm-11-06000-s001.zip › jcm-1960661-supplementary.pdf]

Supplementary Materials

Torii H, Mori K et al. Short-Term Exposure to Violet Light Emitted from Eyeglass Frames in Myopic Children: A Randomized Pilot Clinical Trial

Number of Tables: 8

Titles of Tables:

Table S1. Specifications of VLf.

Table S2. Safety results regarding visual acuity, intraocular pressure, fluorescein corneal and conjunctival staining scores, tear film break-up time, and corneal endothelial cell density.

Tables S3–S5. Results of the mixed-effects model fitted to the 6-month changes in the cycloplegic objective/subjective refractions and axial length in both eyes in the subgroup analysis (second-half cases aged 8 to 10 years; n = 30 eyes comprised of 10 and 20 eyes in the VLf and placebo groups, respectively).

Tables S6–S8. The adjusted mean changes in the cycloplegic objective and subjective refractions and axial length in both eyes at each visit in the subgroup analysis (second half cases aged 8 to 10 years; n = 30 eyes comprised of 10 and 20 eyes in the VLf and placebo groups, respectively).

Table S1. Specifications of VLf.

Irradiation method	Violet light (VL) with a wavelength between 360-400 nm is irradiated into the eyes.<br>VL light-emitting diodes are positioned on both sides of the upper left and right parts of the eyeglass frame, directed toward the face.<br>The irradiation angle from the eyeglass reference line of sight is 53°.<br>Lights are manually turned on and off.
Irradiance reference values	Irradiance reference value for ocular alignment:<br>310 μW/cm² +100%, −50% （VLf trial device),<br>10 μW/cm² +100%, −50% （control device）
Electronic circuits	The device is equipped with charging, power switch, exposure time control, and back-up circuits.<br>The exposure record may be transmitted via bluetooth low energy.
Power supply	Lithium-ion battery<br>Voltage: 3.7 volt (V)<br>Capacitance: 200 milli-Ampere-hour<br>Charged via universal serial bus power supply (including personal computer, AC100V switching power)
Operating environment temperature/humidity	+5 to +40℃, 30 to 90% relative humidity
Storage environment temperature/humidity	−20 to +60℃, 10% to 95% relative humidity
External dimensions	Approximate width (W) 130 to 155 mm x depth (D) 145 to 170 mm x height (H) 36 mm<br><br>Approximate W 140 to 180 mm x D 35 to 45 mm x H 50 mm when folded
Weight	Approximately 36 g

Table S2. Safety results regarding visual acuity, intraocular pressure, fluorescein corneal and conjunctival staining scores, tear film break-up time, and corneal endothelial cell density.

Item	Eye	Visit	VLf						Control						
			Value			Change from Baseline			Value			Change from Baseline			
			N	Mean	SD	N	Mean	SD	N	Mean	SD	N	Mean	SD	*p* Value
UCVA (logMAR)	R	Baseline	21	0.82	0.23	—	—	—	20	0.85	0.28	—	—	—	—
		4 weeks	21	0.89	0.21	21	0.08	0.15	20	0.86	0.22	20	0.01	0.18	0.183
		12 weeks	18	0.77	0.24	18	-0.04	0.18	16	0.89	0.19	16	-0.03	0.16	0.466
		24 weeks	18	0.86	0.21	18	0.05	0.19	15	0.96	0.25	15	0.01	0.21	0.910
		Discontinued	2	1.22	0.00	2	0.11	0.16	3	0.74	0.24	3	0.10	0.17	—
	L	Baseline	21	0.80	0.24	—	—	—	20	0.88	0.21	—	—	—	—
		4 weeks	21	0.85	0.18	21	0.04	0.17	20	0.88	0.18	20	0.01	0.17	0.906
		12 weeks	18	0.84	0.22	18	0.02	0.16	16	0.91	0.16	16	-0.04	0.13	0.747
		24 weeks	18	0.88	0.18	18	0.07	0.18	15	0.95	0.23	15	-0.01	0.22	0.837
		Discontinued	2	1.11	0.16	2	0.15	0.21	3	0.84	0.15	3	0.14	0.15	—
	B	Baseline	42	0.81	0.23	—	—	—	40	0.86	0.25	—	—	—	—
		4 weeks	42	0.87	0.19	42	0.06	0.16	40	0.87	0.20	40	0.01	0.17	0.435
		12 weeks	36	0.81	0.23	36	-0.01	0.17	32	0.90	0.17	32	-0.04	0.14	0.847
		24 weeks	36	0.87	0.19	36	0.06	0.18	30	0.95	0.24	30	0.00	0.21	0.893
		Discontinued	4	1.17	0.11	4	0.13	0.15	6	0.79	0.19	6	0.12	0.15	—
BCVA (logMAR)	R	Baseline	21	-0.09	0.03	—	—	—	20	-0.08	0.03	—	—	—	—
		4 weeks	21	-0.08	0.00	21	0.01	0.03	20	-0.08	0.04	20	0.00	0.03	—*
		12 weeks	18	-0.08	0.00	18	0.01	0.02	16	-0.07	0.02	16	0.01	0.02	—*
		24 weeks	18	-0.06	0.07	18	0.02	0.07	16	-0.07	0.02	16	0.01	0.02	—*
		Discontinued	2	-0.08	0.00	2	0.05	0.07	3	-0.08	0.00	3	0.00	0.00	—*
	L	Baseline	21	-0.09	0.03	—	—	—	20	-0.08	0.02	—	—	—	—
		4 weeks	21	-0.08	0.00	21	0.01	0.03	20	-0.08	0.04	20	0.01	0.04	—*
		12 weeks	18	-0.08	0.00	18	0.01	0.02	16	-0.07	0.02	16	0.01	0.03	—*
		24 weeks	18	-0.06	0.07	18	0.02	0.07	16	-0.07	0.02	16	0.01	0.03	—*
		Discontinued	2	-0.08	0.00	2	0.05	0.07	3	-0.08	0.00	3	0.00	0.00	—*
	B	Baseline	42	-0.09	0.03	—	—	—	40	-0.08	0.03	—	—	—	—
		4 weeks	42	-0.08	0.00	42	0.01	0.03	40	-0.08	0.04	40	0.00	0.04	—*
		12 weeks	36	-0.08	0.00	36	0.01	0.02	32	-0.07	0.02	32	0.01	0.03	—*
		24 weeks	36	-0.06	0.07	36	0.02	0.07	32	-0.07	0.02	32	0.01	0.03	—*
		Discontinued	4	-0.08	0.00	4	0.05	0.06	6	-0.08	0.00	6	0.00	0.00	—*
Intraocular pressure (mmHg)	R	Baseline	21	17.10	2.10	—	—	—	20	17.75	2.53	—	—	—	—
		4 weeks	21	17.10	3.18	21	0.00	2.32	20	17.40	2.50	20	-0.35	2.30	0.802
		12 weeks	18	16.67	3.34	18	-0.33	2.99	16	17.50	2.48	16	-0.63	3.05	0.783
		24 weeks	19	16.68	3.70	19	-0.26	3.11	16	18.25	2.59	16	0.13	2.66	0.646
		Discontinued	2	20.00	5.66	2	1.50	2.12	3	13.00	2.65	3	-2.67	1.53	—
	L	Baseline	21	17.00	3.11	—	—	—	20	17.15	2.56	—	—	—	—
		4 weeks	21	16.10	2.43	21	-0.90	3.11	20	17.35	2.30	20	0.20	1.47	0.067
		12 weeks	18	15.94	3.26	18	-0.89	2.59	16	17.81	2.29	16	0.44	2.19	0.053
		24 weeks	19	16.11	3.28	19	-0.95	2.20	16	17.81	2.69	16	0.44	2.53	0.061
		Discontinued	2	14.50	0.71	2	-2.00	4.24	3	14.33	2.52	3	-0.67	2.08	—
	B	Baseline	42	17.05	2.62	—	—	—	40	17.45	2.53	—	—	—	—
		4 weeks	42	16.60	2.84	42	-0.45	2.75	40	17.38	2.37	40	-0.08	1.93	0.299
		12 weeks	36	16.31	3.28	36	-0.61	2.77	32	17.66	2.35	32	-0.09	2.67	0.254
		24 weeks	38	16.39	3.46	38	-0.61	2.68	32	18.03	2.61	32	0.28	2.56	0.117
		Discontinued	4	17.25	4.57	4	-0.25	3.40	6	13.67	2.42	6	-1.67	1.97	—
Fluorescein	R	Baseline	21	0.1	0.3	—	—	—	20	0.1	0.3	—	—	—	—

corneal and conjunctival staining score		4 weeks	21	0.0	0.0	21	-0.1	0.3	20	0.2	0.4	20	0.1	0.5	0.081
		12 weeks	18	0.1	0.2	18	-0.1	0.2	16	0.2	0.4	16	0.1	0.4	0.198
		24 weeks	19	0.1	0.2	19	-0.1	0.2	16	0.0	0.0	16	-0.1	0.3	0.294
		Discontinued	2	0.0	0.0	2	0.0	0.0	3	0.0	0.0	3	0.0	0.0	—
	L	Baseline	21	0.0	0.2	—	—	—	20	0.1	0.2	—	—	—	—
		4 weeks	21	0.1	0.3	21	0.0	0.2	20	0.1	0.2	20	0.0	0.0	0.372
		12 weeks	18	0.1	0.2	18	0.0	0.3	16	0.1	0.3	16	0.0	0.4	0.951
		24 weeks	19	0.1	0.2	19	0.0	0.0	16	0.0	0.0	16	-0.1	0.3	0.194
		Discontinued	2	0.0	0.0	2	0.0	0.0	3	0.0	0.0	3	0.0	0.0	—
	B	Baseline	42	0.1	0.3	—	—	—	40	0.1	0.3	—	—	—	—
		4 weeks	42	0.0	0.2	42	0.0	0.3	40	0.1	0.3	40	0.0	0.4	0.362
		12 weeks	36	0.1	0.2	36	0.0	0.3	32	0.1	0.3	32	0.0	0.4	0.294
		24 weeks	38	0.1	0.2	38	0.0	0.2	32	0.0	0.0	32	-0.1	0.3	0.097
		Discontinued	4	0.0	0.0	4	0.0	0.0	6	0.0	0.0	6	0.0	0.0	—
BUT (seconds)	R	Baseline	21	7.17	2.61	—	—	—	20	5.96	1.34	—	—	—	—
		4 weeks	21	5.77	1.74	21	-1.40	3.02	20	5.47	1.24	20	-0.49	1.91	0.892
		12 weeks	18	6.11	1.82	18	-1.12	2.22	16	5.03	1.76	16	-1.01	1.62	0.129
		24 weeks	19	5.70	1.64	19	-1.65	2.94	16	5.61	2.39	16	-0.43	2.56	0.816
		Discontinued	2	4.55	0.92	2	-0.95	0.78	3	4.07	1.32	3	-1.30	1.84	—
	L	Baseline	21	6.83	1.74	—	—	—	20	6.07	1.05	—	—	—	—
		4 weeks	21	5.69	1.54	21	-1.15	1.87	20	5.66	1.45	20	-0.41	1.97	0.739
		12 weeks	18	6.02	1.05	18	-0.82	1.62	16	5.51	1.51	16	-0.59	1.56	0.434
		24 weeks	19	5.71	1.17	19	-1.28	1.80	16	5.96	2.05	16	-0.13	2.06	0.416
		Discontinued	2	4.70	0.57	2	-0.65	0.64	3	5.30	2.20	3	-0.53	2.91	—
	B	Baseline	42	7.00	2.20	—	—	—	40	6.01	1.19	—	—	—	—
		4 weeks	42	5.73	1.62	42	-1.27	2.48	40	5.57	1.33	40	-0.45	1.92	0.990
		12 weeks	36	6.06	1.46	36	-0.97	1.92	32	5.27	1.63	32	-0.80	1.57	0.128
		24 weeks	38	5.70	1.41	38	-1.47	2.41	32	5.79	2.20	32	-0.28	2.29	0.634
		Discontinued	4	4.63	0.63	4	-0.80	0.61	6	4.68	1.76	6	-0.92	2.22	—
Corneal endothelial cell density (cells/mm^2)	R	Baseline	21	2895.5	192.0	—	—	—	20	2973.3	200.1	—	—	—	—
		4 weeks	21	2826.2	243.2	21	-69.2	211.4	20	2947.5	228.0	20	-25.8	162.1	0.336
		12 weeks	18	2908.3	200.5	18	23.6	179.9	16	2919.3	313.8	16	-43.6	176.8	0.376
		24 weeks	19	2927.4	223.8	19	28.5	163.1	16	2954.9	248.0	16	-7.9	170.3	0.636
		Discontinued	2	2830.0	186.7	2	-32.5	142.1	3	2828.0	52.8	3	-114.7	186.2	—
	L	Baseline	21	2939.1	223.9	—	—	—	20	2907.0	249.1	—	—	—	—
		4 weeks	21	2920.0	192.2	21	-19.1	150.7	20	2955.4	251.6	20	48.4	139.3	0.153
		12 weeks	18	2876.1	215.8	18	-50.9	127.4	16	2931.2	263.8	16	22.9	109.2	0.160
		24 weeks	19	2923.7	189.4	19	-11.5	105.3	16	2938.7	265.0	16	30.4	174.8	0.536
		Discontinued	2	2931.5	160.5	2	-45.0	104.7	3	2777.0	137.2	3	-81.3	219.2	—
	B	Baseline	42	2917.3	207.2	—	—	—	40	2940.2	225.5	—	—	—	—
		4 weeks	42	2873.1	221.6	42	-44.2	183.1	40	2951.5	237.0	40	11.3	153.8	0.127
		12 weeks	36	2892.2	205.9	36	-13.7	158.2	32	2925.2	285.2	32	-10.3	148.5	0.965
		24 weeks	38	2925.6	204.5	38	8.5	136.9	32	2946.8	252.6	32	11.3	170.9	0.982
		Discontinued	4	2880.8	153.7	4	-38.8	102.2	6	2802.5	97.1	6	-98.0	182.8	—

VLf, violet light eyeglasses frames; N, number of eyes; SD, standard deviation; UCVA, uncorrected visual acuity; logMAR, logarithm of the minimum angle of resolution; BCVA, best-corrected visual acuity; R, right eye; L, left eye; B, both eyes; BUT, tear film break-up time.

* Did not converge.

Tables S3–S5. Results of the mixed-effects model fitted to the 6-month changes in the cycloplegic objective/subjective refractions and axial length in both eyes in the subgroup analysis (second-half cases aged 8 to 10 years; n = 30 eyes comprised of 10 and 20 eyes in the VLf and placebo groups, respectively).

Table S3. Results of the mixed-effects model fitted to the 6-month cycloplegic objective refraction change.

Means	Partial Regression Coefficient	95% CI			*p* Value
Treatment group: control* vs. VLf	0.4700	0.0059	~	0.9340	**0.048**
Eyes: right* vs. left	0.0558	−0.1425	~	0.2541	0.552
Visit weeks: 24 weeks* vs. 4 weeks	0.3953	0.2403	~	0.5503	**<0.001**
Age at baseline (years)	0.0873	−0.1284	~	0.3029	0.396
Sex: male* vs. female	0.3932	−0.5716	~	1.3580	0.394
Parents with myopia: 1 (mother or father)* vs. 2 (both)	−0.4676	−1.0287	~	0.0935	0.095
Cycloplegic objective refraction at baseline (spherical equivalent, diopters)	0.0011	−0.0022	~	0.0044	0.488
Intraocular pressure (mmHg)	0.0021	−0.0051	~	0.0093	0.541
Fluorescein corneal and conjunctival staining score	−0.0297	−0.7562	~	0.6968	0.930
Tear film break-up time (seconds)	−0.0115	−0.1746	~	0.1515	0.880
Time spent in outdoor activity before entering elementary school (minutes/day): 30≤ <60* vs. 60≤ <120	0.3218	−0.2401	~	0.8837	0.236
Time spend tin outdoor activity before entering elementary school (minutes/day): 30≤ <60* vs. 120≤	0.6639	−0.2399	~	1.5677	0.136
Reading distance (cm)	0.0048	−0.0257	~	0.0353	0.738
Time spent sleeping (hours/day)	−0.0041	−0.0147	~	0.0065	0.418
Club activity: indoor* vs. outdoor	0.3723	−0.7790	~	1.5236	0.497
Violet light exposure from outdoor（Joule/cm^2）	0.0129	−0.0188	~	0.0446	0.396
Time spent in near work (minutes/day)	−0.0034	−0.0090	~	0.0022	0.212
Time exposed to sunlight (minutes/day)	−0.0056	−0.0115	~	0.0004	0.066

VLf, violet light eyeglasses frames; CI, confidence interval.
*Reference value.

Table S4. Results of the mixed-effects model fitted to the 6-month cycloplegic subjective refraction change.

Means	Partial Regression Coefficient	95% CI	*p* Value
Treatment group: control* vs. VLf	0.4828	0.1414 ~ 0.8241	**0.008**
Eyes: right* vs. left	−0.0272	−0.1344 ~ 0.0799	0.588
Visit weeks: 24 weeks* vs. 4 weeks	0.2408	0.0868 ~ 0.3949	**0.003**
Age at baseline (years)	0.1345	0.0102 ~ 0.2589	**0.036**
Sex: Male* vs. female	0.1799	−0.3977 ~ 0.7574	0.512
Parents with myopia: 1 (mother or father)* vs. 2 (both)	−0.2625	−0.6169 ~ 0.0918	0.133
Cycloplegic subjective refraction at baseline (spherical equivalent, diopters)	−0.0003	−0.0020 ~ 0.0015	0.752
Intraocular pressure (mmHg)	0.0030	−0.0009 ~ 0.0068	0.117
Fluorescein corneal and conjunctival staining score	0.0873	−0.2987 ~ 0.4733	0.628
Tear film break-up time (seconds)	−0.0347	−0.1234 ~ 0.0540	0.407
Time spent in outdoor activity before entering elementary school (minutes/day): 30≤ <60* vs. 60≤ <120	0.5324	0.1703 ~ 0.8944	**0.008**
Time spent in outdoor activity before entering elementary school (minutes/day): 30≤ <60* vs. 120≤	0.6729	0.0851 ~ 1.2607	**0.028**
Reading distance (cm)	0.0166	0.0002 ~ 0.0330	**0.048**
Time spent sleeping (hours/day)	−0.0006	−0.0064 ~ 0.0052	0.816
Club activity: indoor* vs. outdoor	0.1277	−0.4825 ~ 0.7379	0.659
Violet light exposure outdoors (Joule/cm^2)	0.0049	−0.0116 ~ 0.0214	0.535
Time spent on near work (minutes/day)	−0.0039	−0.0072 ~ −0.0005	**0.029**
Time exposed to sunlight (minutes/day)	−0.0042	−0.0075 ~ −0.0010	**0.016**

VLf, violet light eyeglasses frames; CI, confidence interval.
*Reference value.

Table S5. Results of the mixed-effects model fitted to the 6-month axial length change.

Means	Partial Regression Coefficient	95% CI	*p* Value
Treatment group: control* vs. VLf	−0.0927	−0.1660 ~ −0.0194	**0.016**
Eyes: right* vs. left	−0.0070	−0.0305 ~ 0.0166	0.532
Visit weeks: 24 weeks* vs. 4 weeks	−0.1811	−0.2125 ~ −0.1496	**<0.001**
Visit weeks: 24 weeks* vs. 12 weeks	−0.0955	−0.1231 ~ −0.0679	**<0.001**
Age at baseline (years)	0.0249	−0.0038 ~ 0.0536	0.083
Sex: male* vs. female	−0.1833	−0.3370 ~ −0.0295	**0.023**
Parents with myopia: 1 (mother or father)* vs. 2 (both)	0.1084	0.0419 ~ 0.1748	**0.004**
Axial length at baseline (mm)	−0.0004	−0.0007 ~ 0.0000	0.054
Intraocular pressure (mmHg)	−0.0002	−0.0011 ~ 0.0006	0.574
Fluorescein corneal and conjunctival staining score	−0.0500	−0.1429 ~ 0.0429	0.264
Tear film break-up time (seconds)	0.0058	−0.0135 ~ 0.0251	0.527
Time of outdoor activity before entering elementary school (minutes/day): 30≤ <60* vs. 60≤ <120	−0.0368	−0.1120 ~ 0.0384	0.307
Time of outdoor activity before entering elementary school (minutes/day) : 30≤ <60* vs. 120≤	−0.0906	−0.2029 ~ 0.0218	0.104
Reading distance (cm)	−0.0010	−0.0050 ~ 0.0030	0.604
Time spent sleeping (hours/day)	0.0015	0.0000 ~ 0.0031	0.056
Club activity: indoor* vs. outdoor	−0.1847	−0.3469 ~ −0.0225	**0.029**
Violet light exposure outdoors (Joule/cm^2)	−0.0021	−0.0048 ~ 0.0007	0.132
Time spent in near work (minutes/day)	0.0008	0.0001 ~ 0.0016	**0.039**
Time exposed to sunlight (minutes/day)	0.0010	0.0003 ~ 0.0018	**0.011**

VLf, violet light eyeglasses frames; CI, confidence interval;
*Reference value.

Tables S6–S8. The adjusted mean changes in the cycloplegic objective and subjective refractions and axial length in both eyes at each visit in the subgroup analysis (second half cases aged 8 to 10 years; n = 30 eyes comprised of 10 and 20 eyes in the VLf and placebo groups, respectively).

**Table S6**	**Adjusted Mean Change in Cycloplegic Objective Refraction in both Eyes at Each Visit**		
	Control (n = 20 eyes)	VLf (n = 10 eyes)	
Visit, weeks	Progression, diopters	Progression, diopters	*P* value
4	−0.25 ± 0.14	−0.01 ± 0.17	0.270
24	−0.64 ± 0.15	−0.18 ± 0.17	**0.048***

**Table S7**	**Adjusted Mean change in Cycloplegic Subjective Refraction in both Eyes at Each Visit**		
	Control (n = 20 eyes)	VLf (n = 10 eyes)	
Visit, weeks	Progression, diopters	Progression, diopters	*P* value
4	−0.36 ± 0.09	0.05 ± 0.10	**0.012***
24	−0.60 ± 0.10	−0.12 ± 0.12	**0.008***

**Table S8**	**Adjusted Mean Change in Axial Length in both Eyes at Each Visit**		
	Control (n = 20 eyes)	VLf (n = 10 eyes)	
Visit, weeks	Elongation, mm	Elongation, mm	*P* value
4	0.05 ± 0.01	−0.01 ± 0.02	**0.020***
12	0.14 ± 0.01	0.07 ± 0.02	**0.015***
24	0.23 ± 0.02	0.14 ± 0.03	**0.016***

The data are expressed as the estimated values ± standard errors.

VLf, violet light eyeglasses frames; *Statistically significant.
